# Baroreflex Control of Heart Rate in Mice Overexpressing Human SOD1: Functional Changes in Central and Vagal Efferent Components

**DOI:** 10.1007/s12264-018-0302-y

**Published:** 2018-11-20

**Authors:** Jin Chen, He Gu, Robert D. Wurster, Zixi Cheng

**Affiliations:** 1 Division of Neuroscience and Division of Metabolic and Cardiovascular Sciences, Burnett School of Biomedical Sciences, College of Medicine, University of Central Florida, Orlando, FL 32816 USA; 2 Department of Cellular and Molecular Physiology, Stritch School of Medicine, Loyola University, Maywood, IL 60153 USA

**Keywords:** SOD1, Parasympathetic, Baroreflex

## Abstract

Excessive reactive oxygen species (ROS) (such as the superoxide radical) are commonly associated with cardiac autonomic dysfunctions. Though superoxide dismutase 1 (SOD1) overexpression may protect against ROS damage to the autonomic nervous system, superoxide radical reduction may change normal physiological functions. Previously, we demonstrated that human SOD1 (hSOD1) overexpression does not change baroreflex bradycardia and tachycardia but rather increases aortic depressor nerve activity in response to arterial pressure changes in C57B6SJL-Tg (SOD1)2 Gur/J mice. Since the baroreflex arc includes afferent, central, and efferent components, the objective of this study was to determine whether hSOD1 overexpression alters the central and vagal efferent mediation of heart rate (HR) responses. Our data indicate that SOD1 overexpression decreased the HR responses to vagal efferent nerve stimulation but did not change the HR responses to aortic depressor nerve (ADN) stimulation. Along with the previous study, we suggest that SOD1 overexpression preserves normal baroreflex function but may differentially alter the functions of the ADN, vagal efferents, and central components. While SOD1 overexpression likely enhanced ADN function and the central mediation of bradycardia, it decreased vagal efferent control of HR.

## Introduction

Many cardiovascular diseases are associated with autonomic dysfunctions [1–9]. In particular, one of these is impaired baroreflex control of heart rate (HR). Baroreflex sensitivity is a measure of the strength of baroreflex control of the HR in response to an arterial pressure change, and is an independent index of cardiac autonomic functions. One possible cause of the impaired baroreflex sensitivity may be the excessive generation of reactive oxygen species (ROS) in these diseases [10, 11]. Thus, antioxidant enzymes have been proposed to be potential treatments for impaired autonomic functions induced by sleep apnea and diabetes [12–14]. However, the evidence available from the literature suggests that superoxide dismutase 1 (SOD1) overexpression can have either protective or detrimental effects on tissues [15]. Therefore, we need to consider whether hSOD1 overexpression can affect the neural components of the baroreflex arc in healthy animals. Only then, can we consider any potential benefits of SOD1 overexpression in disease-induced impairment of the baroreflex arc. Previously, we have determined the effects of hSOD1 overexpression in transgenic mice on several physiological variables [arterial pressure (AP), heart rate (HR), baroreflex sensitivity, and aortic depressor nerve (ADN) function] compared to controls [15]. These findings indicated that hSOD1 overexpression in transgenic mice does not alter the values of AP, HR, and baroreflex sensitivity but enhances ADN function. The baroreflex arc includes the vagal aortic depressor nerves, central components (e.g., the nucleus of the solitary tract and vagal motor nucleus), and vagal efferent nerves to the heart. Since hSOD1 overexpression enhances aortic depressor nerve function but maintains baroreflex sensitivity, we hypothesized that hSOD1 overexpression may alter the central mediation and/or vagal efferent control of HR. Indeed, SOD1 overexpression enhanced the central mediation of bradycardia but decreased the vagal efferent control of HR. Along with our previous study, our work provides baseline data on the effects of the hSOD1 overexpression on the baroreflex arc. This will facilitate future studies of the possible protective effects of overexpressed SOD1 in mouse models of disease.

## Materials and Methods

### Animals

Male C57BL/6 J mice of 3–4 months old served as controls for the transgenic Cu/Zn SOD mice (C57B6SJL-Tg (SOD1)2 Gur/J, Jackson catalog #002297). The animal protocols were approved by the University of Central Florida Institutional Animal Care and Use Committee (UCF IACUC) (Animal Project no. 08–50) and followed the guidelines established by the National Institutes of Health Guide for the Care and Use of Laboratory Animals (8th edition, 2011). All experiments were conducted in accordance with the recommendations of the UCF IACUC.

### Surgical Procedure

Mice were anesthetized by 3% isoflurane inhalation and maintained with 1% isoflurane in a mixture of 95% O_2_ and 5% CO_2_ through a tracheal tube. Depth of anesthesia was monitored by eye-blink and withdrawal reflexes (toe-pinch) as well as fluctuations in arterial blood pressure. Body temperature was maintained at 37 ± 1 °C with a homeostatic plate and a rectal probe (ATC 1000; World Precision Instruments, Sarasota, FL). Tapered polyethylene catheters (PE-50) were placed in the left femoral artery to monitor AP and in the right femoral vein to infuse anesthetic agents. Wire electrodes were attached to the legs to record the electrocardiogram (ECG). These mice were used to assess AP and HR, as well as the cardiovascular responses to stimulation of the left ADN or vagal nerve. All experiments were conducted while the animals were maintained in the anesthetized state.

### AP, HR, and Baroreflex Control of HR

The blood pressure catheter was connected to a blood pressure transducer (MIT0699, ADInstruments). The transducer tip was positioned near the level of the heart. AP was measured using the PowerLab Data Acquisition System (PowerLab/8 SP). Mean AP (MAP) and HR were derived from the AP values using Chart 5 software (ADInstruments). ADN activity (ADNA), integrated ADNA (Int ADNA), phasic AP (PAP), and ECG were all recorded and simultaneously displayed on different channels of Chart 5. The methods of calculating AP and HR were identical to those described previously [15, 16].

### HR and MAP Responses to Stimulation of the Left ADN

A cervical midline incision was performed, and the trachea was cannulated (Polyethylene-50) to facilitate ventilation in spontaneously breathing mice. The left ADN was identified in the cervical region under a dissecting microscope and carefully isolated from surrounding connective tissues using fine glass tools to avoid injury of the nerve. Then, the nerve was placed on miniaturized bipolar, platinum electrodes (0.12 mm outer diameter) and covered in mineral oil. The left ADN was then crushed at a point caudal to the electrode to eliminate afferent input to the nucleus of the solitary tract. The baseline values of HR and MAP were measured 30 s prior to stimulation. The nerve was stimulated with rectangular current pulses (3 µA, 1 ms) delivered to the electrode at frequencies of 2–60 Hz by a Grass S48 Stimulator (Grass Instruments, West Warwick, RI) through an isolation unit (Grass model PSIU 6). The duration of the stimulus train was 20 s, with a 3–5 min inter-stimulus interval to allow recovery. HR and MAP responses were measured at least twice in each experiment with the order of changes of frequency reversed during the second round of stimulation. The responses were repeatable. The maximal HR and MAP responses to electrical stimulation of the left ADN were measured. In addition, the time courses of the HR and MAP responses were determined as percentages of the maximum value during stimulation, which were sampled and averaged every 1.0 s. The stimulation-induced changes in HR and MAP were abolished after the left ADN was crushed cranial to the electrode, confirming that the responses were reflex in nature.

### HR and MAP Responses to Electrical Stimulation of the Right Cervical Vagus Nerve

The right cervical vagus nerve was carefully dissected free from surrounding structures and cut just caudal to the nodose ganglion, then the caudal, cut end was placed on a pair of bipolar platinum hook electrodes and electrically stimulated with a Grass Stimulator (S48). The stimuli [square wave pulses (30 μA, 1 ms) at 2–30 Hz for 20 s] were delivered *via* an isolation unit (SIU 6). After each stimulation of the left ADN or right vagal nerve, HR and AP returned to their pre-stimulus baseline levels. Maximal HR and MAP responses to electrical stimulation of the cervical vagal nerve were measured. The data were analyzed as described for ADN stimulation. The stimulation-induced changes in HR and MAP were abolished after the vagus nerve was crushed caudal to the electrode, confirming that the responses were indeed due to vagal efferent activity.

## Statistical Analysis

Data are presented as mean ± SEM. Differences between two groups were determined using Student’s *t*-tests. To compare the differences in HR and MAP curves between C57 and SOD1 mice, two-way analysis of variance (ANOVA) with repeated measures followed by Student–Newman–Keuls *post hoc* tests were used. Statistical significance was set at *P* < 0.05.

## Results

Consistent with Hatcher *et al.* [15], we found that hSOD1 overexpression did not significantly alter baseline MAP and HR (Table 1, *P* > 0.05) and did not change the baroreflex control of HR during sequential sodium nitroprusside and phenylephrine (SNP-PE) administration (data not shown).

**Table 1:** Baseline MAP and HR in C57 and SOD1 mice.

	Average HR (BPM)	MAP (mmHg)
C57 (*n* = 8)	548.15 ± 9.76	86.15 ± 2.77
SOD1 (*n* = 8)	531.78 ± 15.58	86.91 ± 15.58

### SOD1 Overexpression Did Not Change HR and MAP Responses to Aortic Depressor Nerve Stimulation

**Fig. 1:**
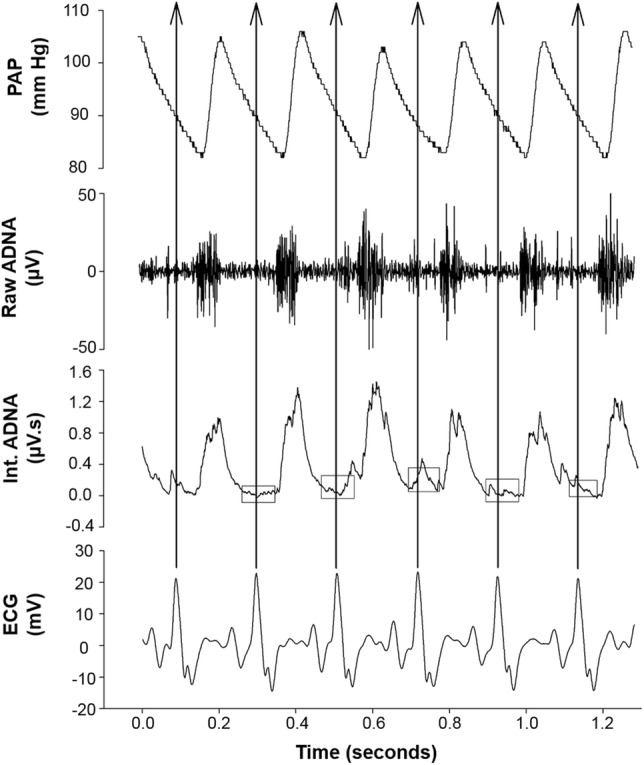
Recordings of phasic arterial pressure (PAP), aortic depressor nerve activity (Raw ADNA), integrated ADNA (Int ADNA), and ECG. Uppermost trace, PAP. Upper middle trace, ADNA occurred as rhythmic bursts that were synchronized with the PAP. Note that ADNA increased before the PAP increases because the PAP catheter was inserted into the femoral artery while ADNA was recorded from the ADN that innervates the aortic arch. Lower middle trace, integrated ADNA using a 10-ms time constant; small boxes enclose silent intervals (noise level) between the ADNA bursts that were used as basal activity. Lowest trace, ECG. The QRS waves were used to automatically separate ADNA firing intervals (arrows).

**Fig. 2:**
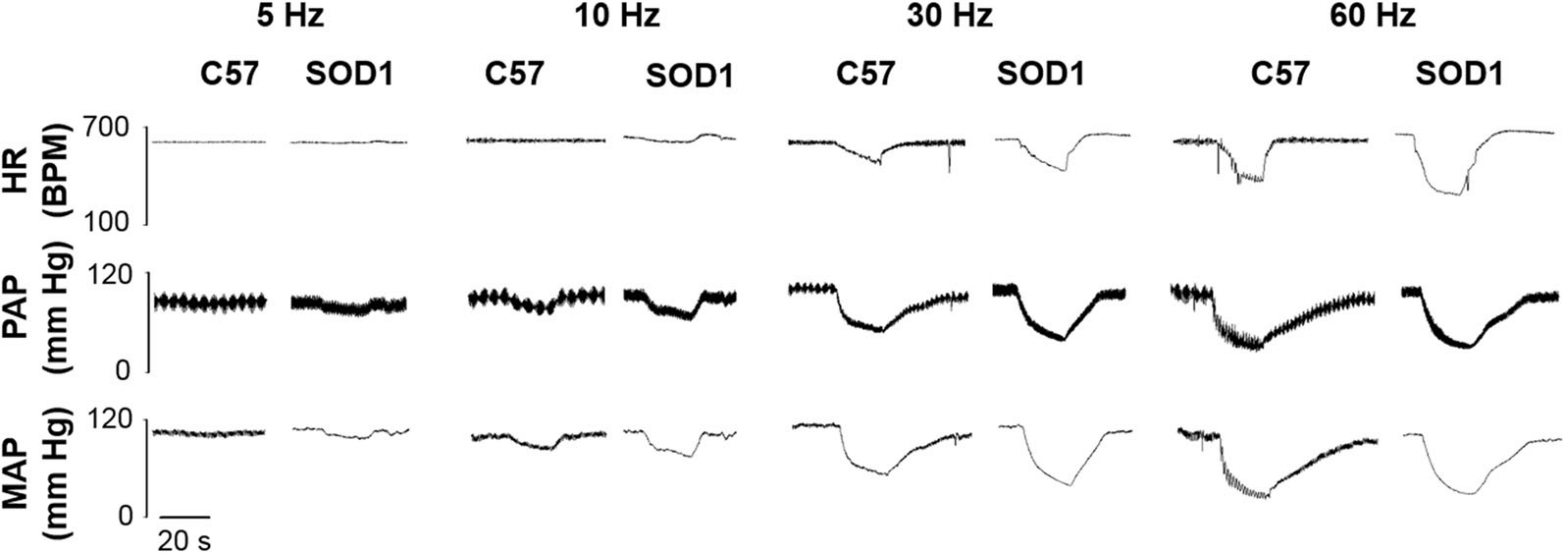
Original recordings of heart rate (HR) depression following stimulation of the aortic depressor nerve at 3 µA, 1-ms pulses in a 20-s train in C57 and SOD1 mice. Representative figures encompass the mid-range frequencies between 5 and 60 Hz.

**Fig. 3:**
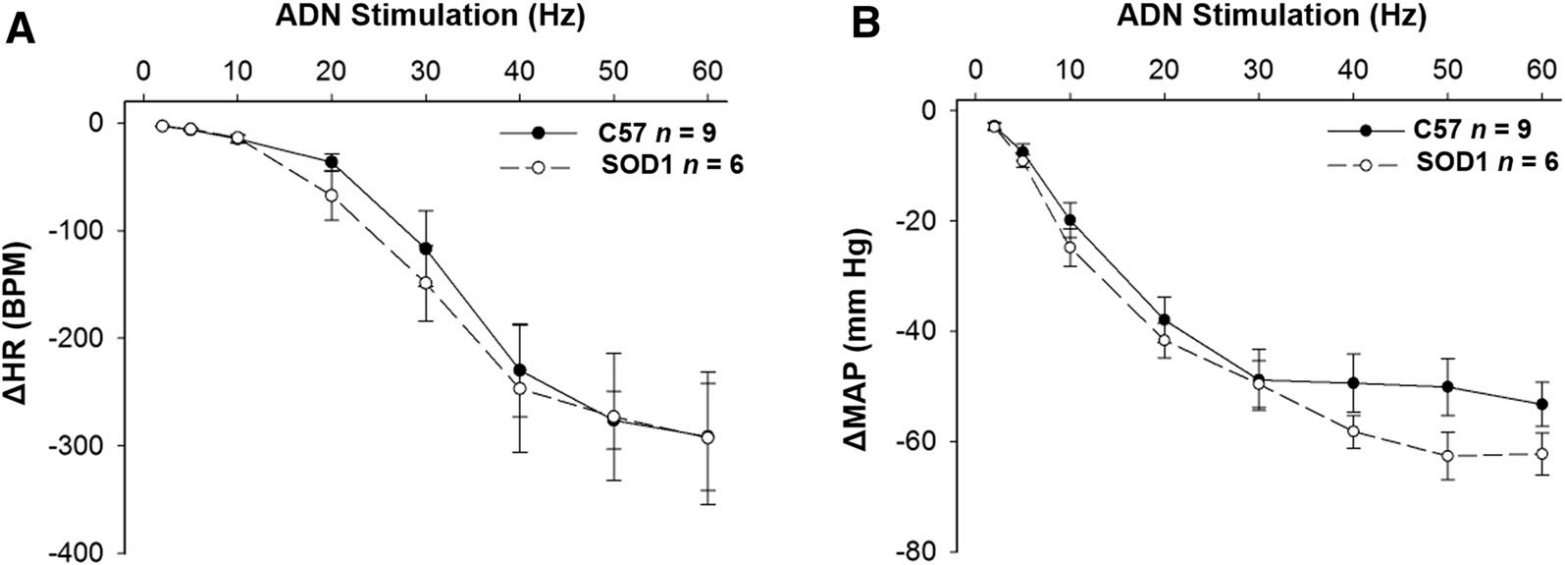
Central mediation of baroreflex bradycardia and mean arterial pressure. Electrical stimulation of the left aortic depressor nerve (ADN) evoked frequency-dependent decreases in HR (**A**) and MAP (**B**) in both C57 and SOD1 mice. They were not significantly different (*P* > 0.05).

**Fig. 4:**
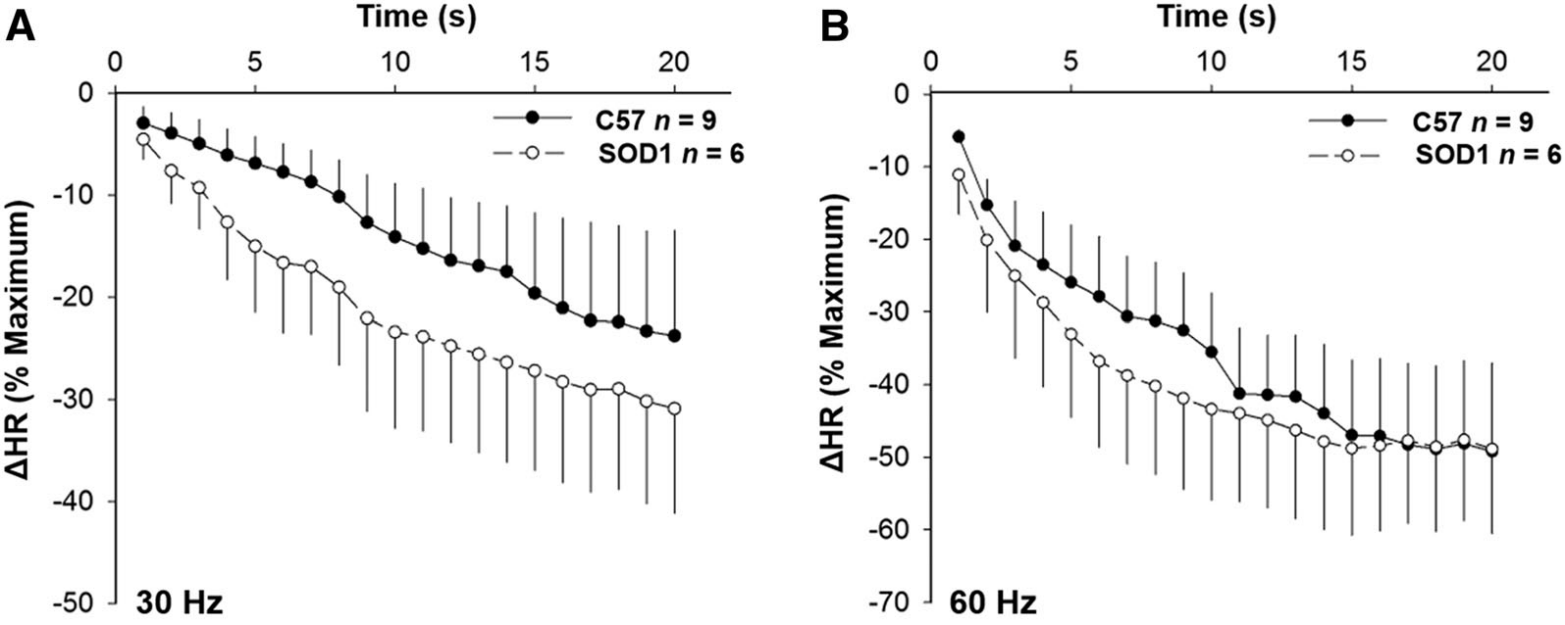
Time courses of HR depression in response to electrical stimulation at 30 (**A**) and 60 Hz (**B**) did not differ between SOD1 and C57 control mice (*P* > 0.05).

After identifying the left ADN (Fig. 1), electrical stimulation evoked frequency-dependent decreases in HR and MAP in SOD1 and C57 mice (Fig. 2). There was no difference in the HR reduction at different frequencies between SOD1 and C57 mice (Fig. 3A). For MAP, there was a slight, but non-significant difference between SOD1 and C57 mice (Fig. 3B). In the time course analysis at 30 and 60 Hz, the HR and MAP responses were not significantly different between the two groups, further indicating that SOD1 overexpression did not change the HR and MAP responses to ADN stimulation (Fig. 4).

### SOD1 Overexpression Decreased HR and MAP Responses to Vagal Efferent Nerve Stimulation

**Fig. 5:**
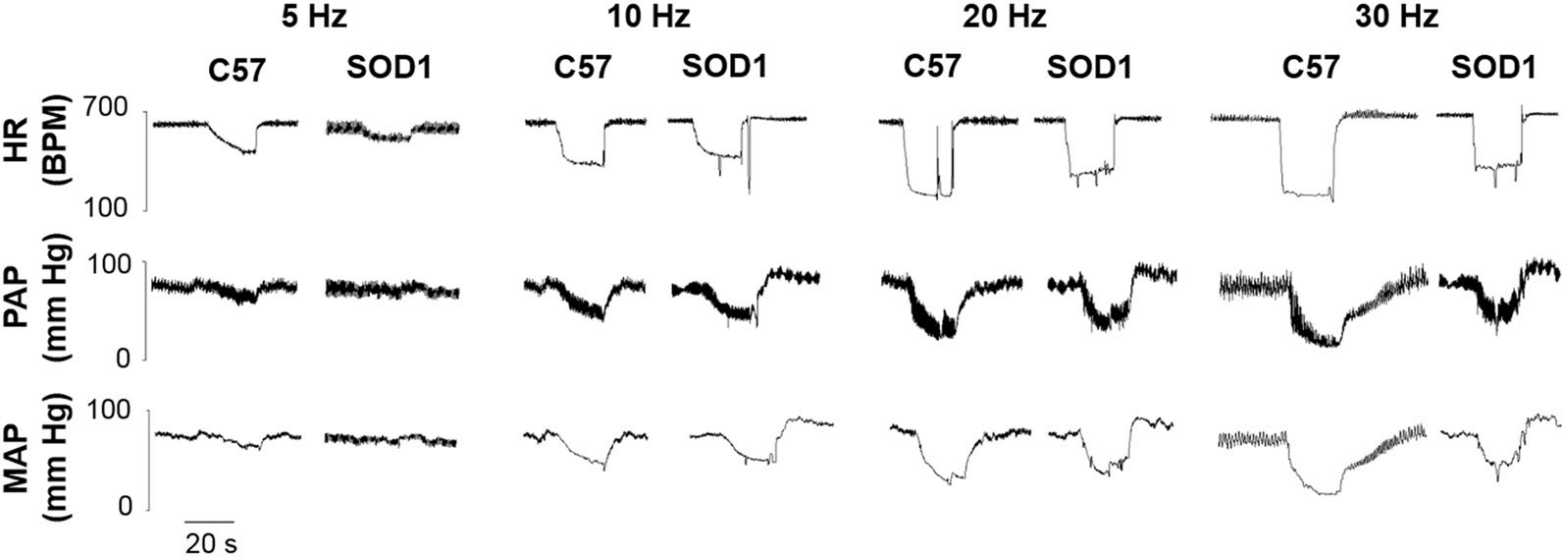
Original recordings of heart rate (HR) depression following stimulation of the right cervical vagus nerve at 30 µA, 1-ms bursts of 20-s trains in C57 and SOD1 mice. Representative figures encompass the mid-range frequencies between 5 and 30 Hz.

**Fig. 6:**
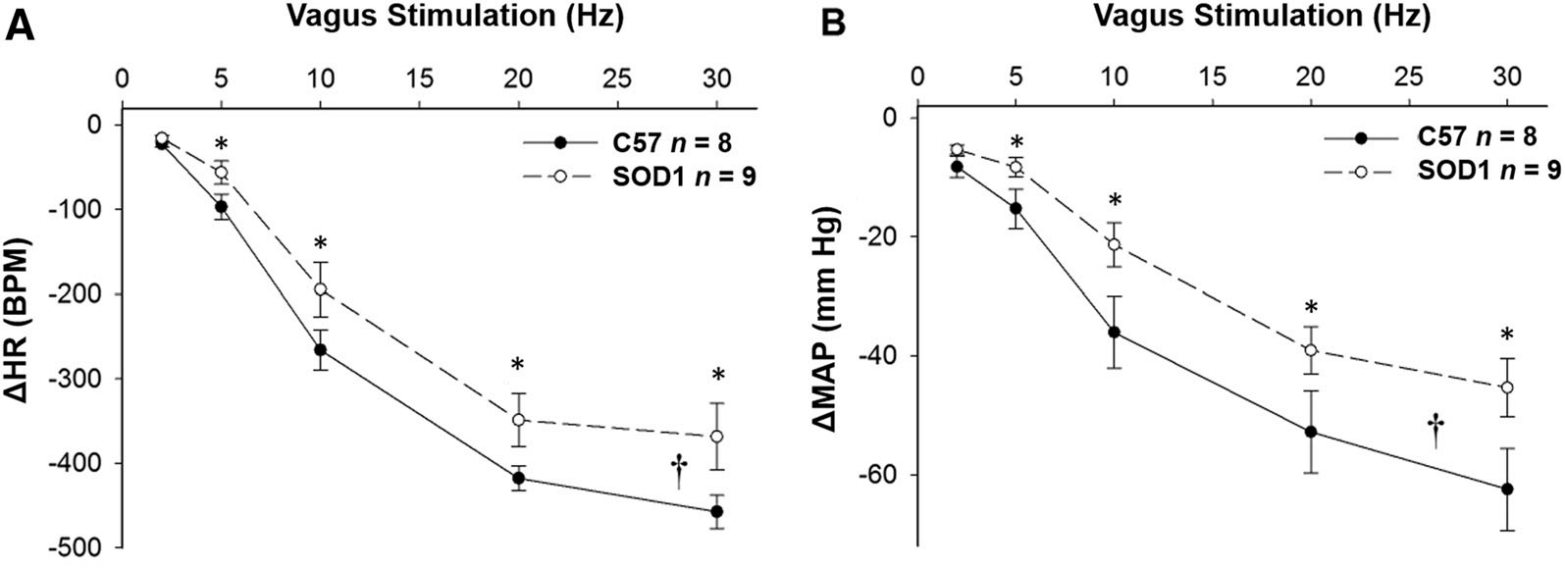
Electrical stimulation of the right cervical vagus nerve evoked frequency-dependent decreases in HR (**A**) and MAP (**B**) in both SOD1 and C57 mice. The differences were significant (**†***P* < 0.05 between groups, *P < 0.05 at the given frequency).

**Fig. 7:**
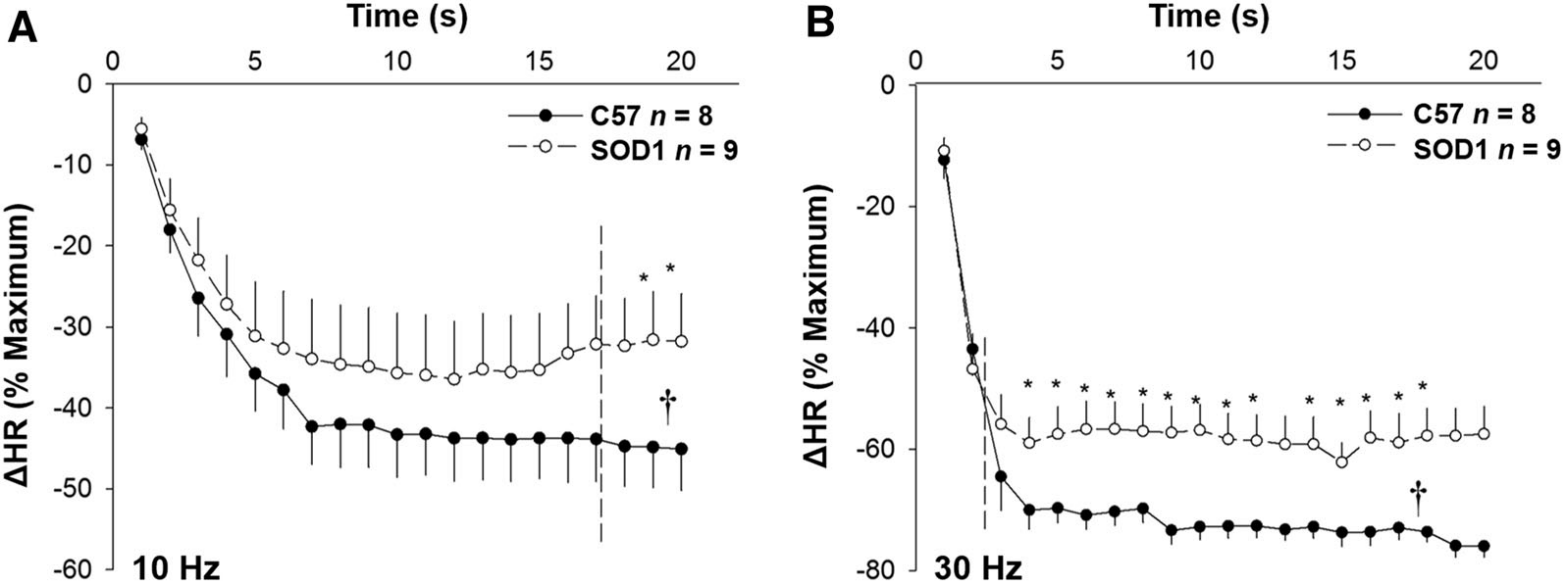
Time courses of HR depression in response to electrical stimulation at 10 (**A**) and 30 Hz (**B**) were significantly different between SOD1 and C57 control mice (^**†**^*P* < 0.05 between groups, *P < 0.05 at a given time).

Electrical stimulation of the vagal efferent nerve evoked frequency-dependent decreases in HR and MAP in both SOD1 and C57 mice (Fig. 5). There were significant differences in the HR and MAP depressions in response to stimulation at different frequencies between SOD1 and C57 mice (Fig. 6, *P* < 0.05). In addition, in the time course analysis for 10 and 30 Hz, the HR and MAP responses to stimulation of the vagal efferent nerve were significantly different between the two groups at several time points (Fig. 7). While HR is directly controlled by the vagal nerve, the major factor for the rapid MAP drop is possibly the rapid HR reduction in response to vagal stimulation.

## Discussion

In this study, we demonstrated that SOD1 overexpression decreased the HR response to vagal efferent nerve stimulation but did not change the HR response to ADN stimulation. Previously, we have shown that ADN function, i.e., the ADN activity in response to MAP changes, is enhanced, but the baroreflex control of HR is unchanged [15]. Since the baroreflex arc includes the ADN and the central and vagal efferent components, we reasoned that the central mediation of bradycardia must be increased. Along with the previous study [15], we suggest that SOD1 overexpression preserves normal baroreflex sensitivity but may differentially alter the functions of the ADN, vagal efferent, and central components. While SOD1 overexpression enhanced ADN function and the central mediation of bradycardia, it decreased vagal efferent control of the HR.

Previously, we found that the basal AP and HR were not changed in mice overexpressing hSOD1 compared to normal controls. In addition, hSOD1 overexpression did not change the baroreflex control of HR. However, hSOD1 overexpression augmented ADN function [15]. The baroreflex arc includes the ADN and vagal efferents, as well as the central components, which include the nucleus of the solitary tract, nucleus ambiguus, dorsal motor nucleus of the vagus nerve, paraventricular nucleus of the hypothalamus, caudal ventrolateral medulla, and rostral ventrolateral medulla [17–20]. Thus, we reasoned that the increased signaling from the ADN nerves to the brainstem is buffered by other neural components of the baroreflex circuitry. i.e., hSOD1 overexpression may alter either central mediation and/or vagal efferent control of HR. In this study, we first examined whether hSOD1 overexpression altered the HR reduction in response to ADN stimulation. Our data indicated that hSOD1 overexpression did not alter baroreflex bradycardia in response to ADN stimulation. Following up, we tested whether hSOD1 overexpression altered the HR reduction in response to vagal efferent nerve stimulation. We found that hSOD1 overexpression decreased the bradycardia in response to vagal efferent stimulation. Taken together, these findings have revealed that SOD1 overexpression may have different effects on different neural components in the baroreflex circuitry: it increased ADN function and central mediation of reflex bradycardia, but decreased the bradycardia response to vagal efferent stimulation. Although we do not know the exact mechanisms, it is assumed that increased aortic compliance, increased vagal motor output from the brainstem, and reduced cardiac vagal efferent presynaptic function and/or reduced responses of cardiac ganglionic neurons, may account for such differential changes in these neural components in SOD1 mice. Since this is a universal hSOD1 expression model, the other factors (such as vascular walls and heart) are also likely to contribute to functional changes of these neural components.

Previous studies have shown that antioxidants can be used to effectively reduce hypertension, increase vascular compliance, and improve baroreflex-mediated control of heart rate [21–27]. Since ROS have a wide variety of essential biological functions, it is important to *first* evaluate the effects of systemic application of antioxidants on cardiac autonomic functions in an animal model without any diseases. The current study used a transgenic mouse model expressing human Cu/ZnSOD (SOD1) at a level roughly 3.5-fold over normal mouse SOD1 expression in cortical tissue [28]. The hSOD1 mouse line in this study harbors the human mini-SOD1 gene and is expected to express the hSOD1 transgene similar to the endogenous mouse SOD1. As SOD1 is ubiquitously expressed in vertebrates, its expression and activity are expected to be higher in the peripheral tissues of this transgenic line. At this moment, however, we cannot interpret the exact cellular and molecular mechanisms for such an enhancement of ADN function and central mediation as well as the reduction of vagal efferent control of HR. It appears that overexpressing hSOD1 may have different effects on different neural components. Thus, we propose that hSOD1 treatments may very likely have different effects on autonomic neuropathy, depending upon the location of treatments. Our study used this mouse line as a relevant model for exploring the effect of enhanced SOD1 expression on physiological functions, providing an important clue for future mechanistic cellular and molecular studies using this model.

It has been shown that chronic intermittent hypoxia (CIH) and diabetes reduce the central mediation of reflex bradycardia and induce cardiac vagal motor neuron death in the nucleus ambiguus [16, 29–32]. Xu *et al.* [33] used this hSOD1 mouse line and showed that overexpressing hSOD1 increases resistance to oxidative stress and the apoptosis of cortical neurons after exposure to CIH as compared to wild-type controls. Furthermore, hSOD1 overexpression has also been shown to protect against mitochondrial cytochrome C release and subsequent apoptosis in focal cerebral ischemia models of stroke [34]. Since hSOD1 overexpression may increase the central mediation of reflex bradycardia, whether overexpressing hSOD1 may prevent impaired central mediation of reflex bradycardia in CIH and diabetes is an interesting issue and should be tested in the future.
